# A Study of the Lossy Mode Resonances during the Synthesis Process of Zinc Telluride Films

**DOI:** 10.3390/s22218108

**Published:** 2022-10-22

**Authors:** Petr I. Kuznetsov, Dmitriy P. Sudas, Evgeny A. Savelyev

**Affiliations:** 1 Kotel’nikov Institute of Radioengineering and Electronics of the Russian Academy of Sciences (Fryazino Branch), sq. Vvedenskogo 1, Fryazino 141190, Russia; 2 World-Class Research Center, Peter the Great St. Petersburg Polytechnical University, Polytechnicheskaya Ul.29, St. Petersburg 195251, Russia

**Keywords:** lossy mode resonance, optical fiber sensor, refractometer, ZnTe thin film, metalorganic chemical vapor deposition

## Abstract

Films of zinc telluride (ZnTe) were deposited on the surface of a chemically thinned section of an optical fiber by metalorganic chemical vapor deposition. The boundary values of temperatures and the concentration ratios of the initial tellurium and zinc precursors at which the synthesis of ZnTe coatings is possible are determined. The influence of the position of the thinned part of the optical fiber in the reactor on the growth rate of films on the side surface of the fiber was studied, on the basis of which, the parameters of the deposition zone were determined. By placing a section of an optical fiber with an etched cladding in the center of this zone, sensitive elements for refractometers were created. The principle of their operation is based on the dependence of the spectral position of the lossy mode resonance (LMR) maximum on the refractive index (RI) of the external medium. It has been found that even thin films deposited on a light guide in a continuous process have cracks. It is shown that the interruption of the deposition process makes it possible to avoid the appearance of defects in the zinc telluride layers even with the repeated deposition of the sensor. The sensitivity of the spectral position of the LMR to changes in the RI of aqueous sodium chloride solutions in the range from 1.33 to 1.35 for the first transverse electric and transverse magnetic LMRs was 6656 and 6240 nm per refractive index unit, respectively.

## 1. Introduction

For more than six decades since the appearance of the term “fiber optics”, this field has become one of the most popular, not only in optics but also in physics in general [1]. Currently, one of the most rapidly developing areas in this domain is dedicated to fiber-optic sensors (FOSs) [2]. Resistance to external electromagnetic fields, physical and chemical durability, the small transverse size and linear density of the optical fiber, and most importantly, the possibility of the remote monitoring of almost any parameter of the environment make such sensors indispensable devices in many areas of modern life [3,4,5,6,7,8,9]. Quite often, as sensitive elements for FOS, an optical fiber with a coating on the lateral surface is used, which makes it possible to realize in them such phenomena as surface plasmon resonance (SPR) [10,11,12,13,14] and lossy mode resonance (LMR) [15,16,17,18,19]. To detect both types of resonances, it is necessary that the real part of the permittivity of the coating material deposited on the surface of the optical fiber exceeds the same parameter for the light guide and is greater than the absolute value of the imaginary part of the dielectric constant of the film substance [20]. If the real part of the layer permittivity is negative, then SPR is observed, and in the opposite case, LMR is observed. Thus, if metals are the main class of materials utilized for the synthesis of thin films for the realization of the SPR phenomenon, then a wide class of substances such as polymers, semiconductors, and dielectrics is suitable for the observation of LMR. At the same time, if in the case of SPR, it is feasible to recognize only a resonance associated with the excitation of the transverse magnetic (TM) wave of a thin film, then, for LMR, it is possible to register ones of many orders of both types of polarization [21].

To enhance the interaction between the light propagating inside the optical fiber core and the coating material, light guides are usually utilized, the geometry of which is changed within a small section by lateral polishing [22], thermal stretching [23], or chemical thinning [24]. The latter approach has a number of advantages. Firstly, the geometry of the fiber core remains intact, which allows to almost completely avoid the appearance of losses with this processing method [25]. Secondly, the constant distance between the coating material and the fiber core does not require the optimization of the film geometry, as, for example, in the case of D-shaped fibers [26].

The most common application of LMR-based FOS is the fabrication of refractometers [7]. One of the promising coating materials for this purpose may be zinc telluride (ZnTe). It has astonishing optical properties: a high real part of the refractive index (RI) [27] with a strong influence of synthesis conditions on the value of the imaginary part of RI, which makes it feasible to achieve high sensitivities of sensors with such a coating and to control the resonance shape. In the case of using the metalorganic chemical vapor deposition (MOCVD) technology, the relatively low deposition temperature [28] provides an opportunity to prevent the saturation of the dielectric waveguide in a hydrogen atmosphere with a large amount of OH groups. Another advantage of this material is the simplicity of its chemical removal from the surface of amorphous silicon dioxide. This enables the same thinned optical fiber to be repeatedly applied in different experiments, which is very convenient, since the length and outer diameter of the etched section may slightly differ from specimen to specimen, thus affecting the resonance shape.

In this work, the influence of the synthesis conditions of ZnTe thin films on the characteristics of LMR-based fiber-optic refractometers is investigated. To this end, a large series of experiments was performed demonstrating the correlation of resonance shape on parameters such as temperature, the ratio of the initial precursors, and the position of the etched part of the fiber in the MOCVD reactor. A method of stress relief in the film is shown, which allows for avoiding its physical destruction. The sensitivities and figures of merits of the first transverse electric (TE) and TM LMRs are compared.

## 2. Materials and Methods

A standard single-mode fiber (Corning SMF-28) with a cladding/core diameter of 125/8.2 μm was thinned using a low-toxic polishing etchant, which is an aqueous solution of fluoride (NH_4_F) and ammonium sulfate ((NH_4_)_2_SO_4_) (Merck KGaA, Darmstadt, Germany). To improve the surface quality of the etched region and reduce optical losses, we utilized the previously presented rocking method [29]. In this study, light guides with different lengths of the thinned area were fabricated. The first optical fiber, with a shorter etched length (≈2.0 mm), was applied to characterize the deposition zone in the MOCVD reactor. This made it feasible to more accurately detect variations in the parameters of the material changing along the deposition zone. The second fiber, with a longer thinned section (≈3.5 mm), was utilized to manufacture sensitive parts of fiber optic refractometers, which made it easier to extract optical fibers with a thin ZnTe film synthesized on its surface without violating the layer integrity. The outer diameter of the thinned part in both series of experiments was 24.5 ± 1.5 μm.

**Figure 1:**
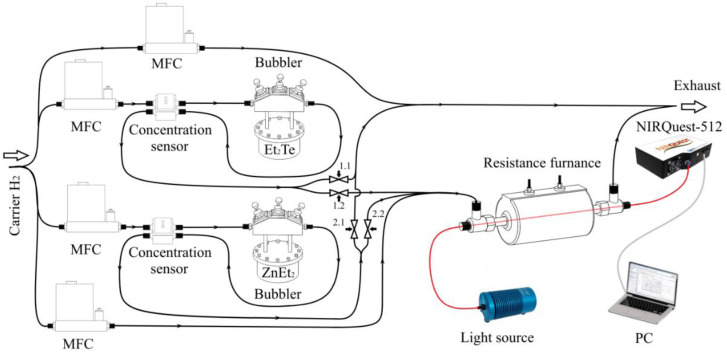
The scheme of deposition of ZnTe films on the lateral surface of an optical fiber by the MOCVD method with parallel control of the transmission spectra of a dielectric light guide.

The deposition of ZnTe films on the lateral surface of the fiber light guide was performed by the MOCVD method. The manufactured optical fiber with a thinned section was placed in a tubular silica glass reactor with an inner diameter of 5 mm and sealed at both ends (Figure 1). We used diethylzinc (ZnEt_2_) and diethyl telluride (Et_2_Te) (Merck KGaA, Darmstadt, Germany) as initial reagents. The coating process was carried out at atmospheric pressure in a stream of thoroughly dried hydrogen with a linear velocity of 15 to 25 cm/s. Using a resistive furnace, the temperature inside the reactor varied in the range from 230 to 320 °C. Before the start of the process, the pneumatic valves 1.1 and 2.1 (Figure 1) are in the open state, and valves 1.2 and 2.1 are in the closed state, which excludes the possibility of the starting reagents entering the reaction zone until the concentration of precursors and the temperature in the reactor reach the required values. There are two lines in the system through which hydrogen is constantly passed, bypassing the bubblers. The carrier gas supplied directly to the cylindrical reactor determines the absolute concentration of reagents in the vapor–gas mixture, and the magnitude of its flow allows for adjusting the position of the deposition zone along the axis of the furnace. A constant flow of hydrogen supplied through the line to the exhaust prevents the possible penetration of the external environment into the deposition zone and also balances the stream going into the reactor. To initiate the process, the pneumatic valves are switched in such a way that the first pair (1.1 and 2.1) is closed and the second pair (1.2 and 2.2) is opened simultaneously.

**Figure 2:**
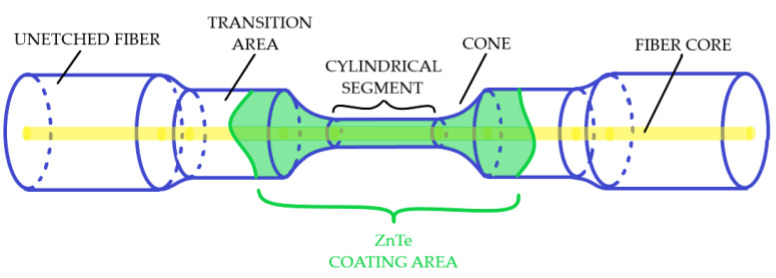
Scheme of a thinned section of a fiber light guide.

During the deposition process, the transmission spectra of the fiber light guide were monitored and recorded every 0.2 s in the wavelength range of 900–1700 nm. An HL-2000 halogen lamp and a NIRQuest-512 (OceanOptics Inc., Rochester, NY, USA) spectrometer (wavelength resolution = 3.1 nm) served as a radiation source and a receiver, respectively. A diagram of a thinned section of a coated fiber is shown in Figure 2.

The surface of all deposited ZnTe coatings was examined for defects on an optical microscope with a digital camera. A number of samples were also examined with an electron microscope JSM-6480LV (Jeol Ltd., Tokyo, Japan) with a tungsten thermionic cathode. To determine the elemental composition of the grown films, we used an energy-dispersive spectrometer X-MaxN, which was docked with the electron microscope. To exclude the influence of changing the geometry of the optical fiber thinned part on the resonance shape, the same light guide was utilized in each series of experiments. This was achieved by removing the examined coating in a mixture of hydrochloric acid and hydrogen peroxide.

## 3. Results and Discussion

### 3.1. Dependence of the Resonance Shape on the Ratio of the Concentrations of the Initial Reagents

At the first stage of the experiments, the minimum and maximum values of the C_Te_/C_Zn_ ratio (C_Te_ and C_Zn_ are the concentrations of Et_2_Te and ZnEt_2_, respectively) and the temperature in the reaction zone were determined, within which, ZnTe deposition on the optical fiber surface was possible. It was preliminarily established that the minimum temperature in the center of the reactor, at which zinc telluride is synthesized at a rate acceptable for practical purposes, is 234 °C. However, extracting a thinned fiber with a ZnTe film deposited at such a low temperature from the reactor without breaking it turned out to be an extremely difficult task. On the other hand, at temperatures of 310 °C and above, a parallel reaction of the decomposition of the synthesized material by hydrogen actively proceeds according to the equation: ZnTe + H_2_ = Zn + H_2_Te, and the rate of ZnTe deposition drops to almost zero. Therefore, further experiments to optimize the synthesis conditions were carried out at a temperature equal to the average value from these extreme points.

**Figure 3:**
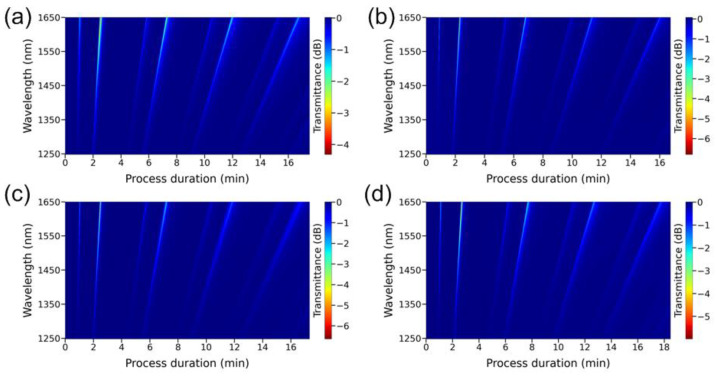
Spectral response in the wavelength range of 1250–1650 nm depending on the duration of the synthesis process of ZnTe film on the surface of a thinned optical fiber at the C_Te_/C_Zn_ ratio equal to: (**a**) 1.13, (**b**) 1.38, (**c**) 1.49, (**d**) 1.80.

By placing the thinned part of the fiber in the middle point of the deposition zone at a temperature in the center of the furnace equal to 272 °C, the C_Te_/C_Zn_ ratio at the reactor inlet varied in a wide range. To do this, at a constant amount of supplied ZnEt_2_, the concentration of Et_2_Te was varied. It appeared that the process of ZnTe film growth occurs only if the C_Te_/C_Zn_ value is in the range from 1.0 to 2.4. Under other conditions, synthesis on the light guide is practically not observed due to the complete blocking of the surface by one of the two initial components. Figure 3 shows the spectral response depending on the duration of the deposition process for four experiments with a value of C_Te_/C_Zn_ from 1.13 to 1.8. It can be seen that the change in the ratio of reagent concentrations within these limits practically does not affect the rate of film deposition. At the same time, this growth parameter has a significant effect on the shape and depth of LMRs.

**Figure 4:**
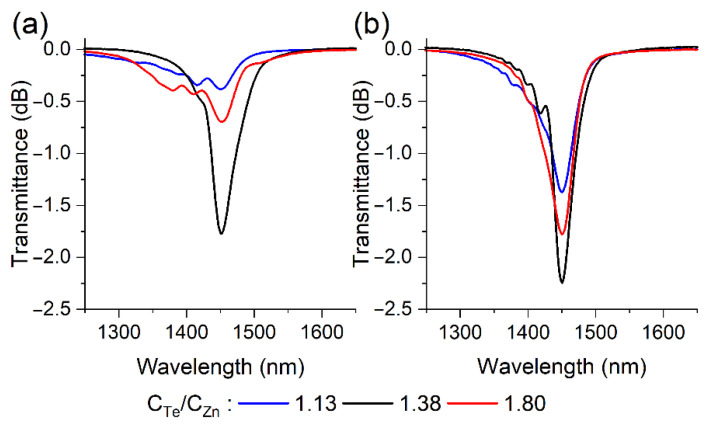
Transmission spectra of thinned optical fibers coated with thin ZnTe films for (**a**) TE and (**b**) TM LMRs at different C_Te_/C_Zn_ ratios.

**Figure 5:**
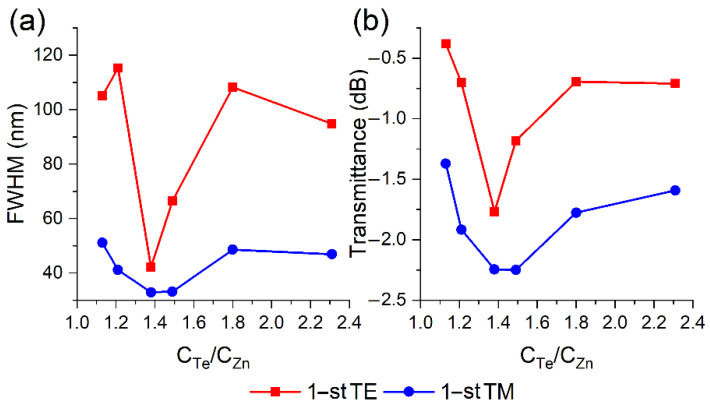
Dependence of (**a**) FWHM and (**b**) peak transmission on the C_Te_/C_Zn_ ratio for the first TE and TM resonances with a maximum at a wavelength of 1450 nm.

Figure 3 shows the transmission spectra of the optical fiber during the observation of the maxima of the first TE (Figure 4a) and TM (Figure 4b) LMRs at a wavelength of 1450 nm, which is the center of the operating spectral range of the SMF-28 fiber. Resonances caused by the interaction of the fundamental mode ($HE_{1,1}$) of the optical fiber with the TE and TM film ones are denoted as TE and TM LMRs, respectively. It is clearly seen from the presented results that TM resonances are deeper, narrower and less dependent on the C_Te_/C_Zn_ ratio than TE resonances. Consequently, the parameters of the resulting zinc telluride film are such that in all resonance orders, a stronger interaction of the fundamental mode with the TM coating mode is observed. To determine the optimal conditions for the synthesis of ZnTe films in terms of the shape of the resonances, the dependences of the transmittance at the resonance maximum wavelength and full width at half maximum (FWHM) were plotted on the ratio of the initial reagents (Figure 5). It is clearly seen from the obtained results that the value of 1.38 is the most suitable one for both types of LMRs from the whole series of the studied relations, which will be utilized in all subsequent experiments in this work.

### 3.2. Determination of Deposition Zone Parameters

**Figure 6:**
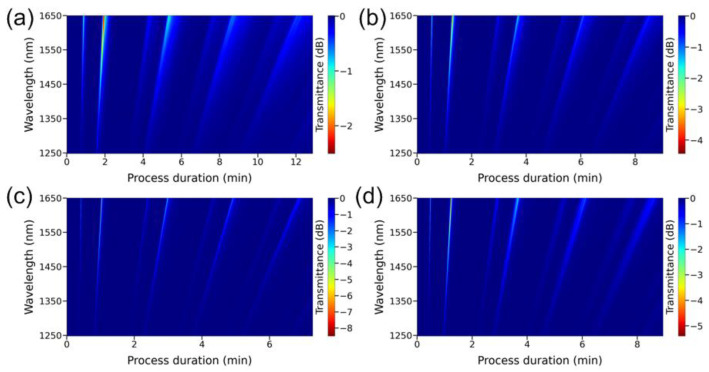
Dependence of the spectral response on the deposition process duration in the wavelength range from 1250 to 1650 nm. The etched region of the optical fiber was located at a distance of (**a**) 0 mm, (**b**) 6 mm, (**c**) 14 mm, and (**d**) 20 mm from the furnace center.

At the next stage, we studied the influence of the position of the etched part of the optical fiber relative to the furnace center on the deposition rate of the ZnTe film in this region. In MOCVD technology, as in almost any other approach used for thin film growth, there is such a term as the deposition zone, within which the coating is synthesized. For the method we exploit, it is determined by two key factors: the change in temperature and the ratio of the concentrations of the used precursors along the reactor, which leads to different deposition rates (Figure 6). The position of the thinned section with a length of 2.0 mm and a diameter of 25.5 μm was measured from the furnace center in the gas flow direction and varied from 0 to 20 mm. The correspondence of the chemical composition of all films deposited on the surface of the optical fiber to zinc telluride was confirmed by analysis on an EDX spectrometer.

**Figure 7:**
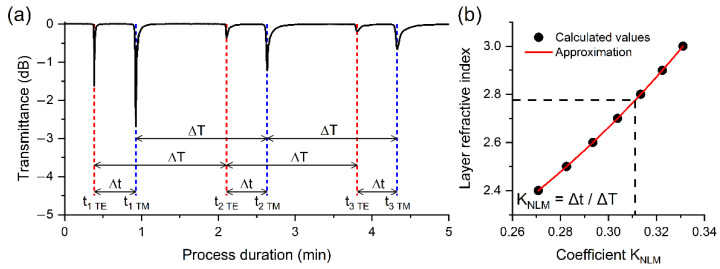
(**a**) Dependence of optical transmission at a wavelength of 1450 nm on the duration of the deposition process, the spectral response of which is shown in Figure 6c. (**b**) Dependence of the coating RI at a wavelength of 1450 nm on the coefficient *K_NLM_*.

To determine the center of the deposition zone and the growth rate ($V_{gr}$), we have previously calculated the RI of the coating. The Neighboring LMR Method (NLM) was applied for this purpose [30]. Let m be the order of resonance, then $t_{m\;TE}$ and $t_{m\;TM}$ are the time points at which TE and TM resonances of the mth order are detected, respectively. It has been demonstrated that for the considered type of dielectric waveguides, LMRs are observed repeatedly [31,32]: $\Delta t=t_{m\;TM}-t_{m\;TE}$, $\Delta T=t_{m\;TE}-t_{m-1\;TE}=t_{m\;TM}-t_{m-1\;TM}.$ If the growth rate of the coating remains constant during the entire process, then $\Delta t/\Delta T=\Delta d/\Delta D=K_{NLM}$, where $d=V_{gr}t$ is the layer thicknesses synthesized over time t. While Δt and ΔT are found from the experiment (Figure 7a), the values of Δd and ΔD can be computed by the finite element method (FEM) [30]. Moreover, the value of ΔD can be calculated with a high level of precision using the following equation [31]:

(1) $$\Delta D=\frac{\lambda_{res}}{2\cdot \sqrt{\left(n_{film}{}^{2}-n_{fiber}{}^{2}\right)}}$$

where $\lambda_{res}$ is the wavelength of the resonance maximum, $n_{film}$ and $n_{fiber}$ are the refractive indices of the coating and optical waveguide (silica glass [33]), respectively. In addition, in order to accurately identify resonances, we carried out theoretical simulations of the positions of resonances, which were correlated with fixed dips in the transmission spectra [31].

**Figure 8:**
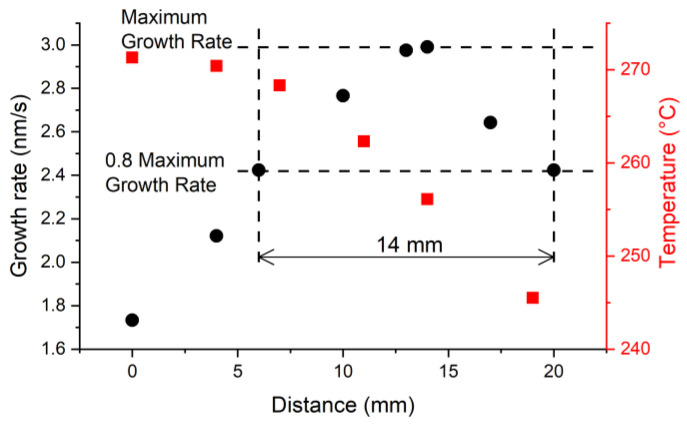
The temperatures inside the reactor (red squares) and the growth rate of the ZnTe film on the surface of the optical fiber (black circles) depending on the position of the thinned area of the light guide relative to the furnace center.

Assuming that the real part RI of the film material does not depend on the position of the thinned region in the reactor, we performed calculations for only one sample with the sharpest resonances and the maximum deposition rate (Figure 6c). Calculations were performed in the operating wavelength center of the SMF-28 fiber at 1450 nm. The obtained value, 2.78 (Figure 7b), is only 1.5% higher than the value measured for a ZnTe crystal at room temperature [27]. It is found that the middle point of the deposition zone is shifted by 14 mm from the reactor center in the direction of the gas stream, while the growth rate changes by no more than 20% in the area with a length of 14 mm (Figure 8). It was not possible to measure the deposition rate at a distance of more than 20 mm due to the breakage of the thinned region during the last experiment. It should be noted that in the experiments in which the etched part was located in a region significantly remote from the deposition zone center in the direction of the gas flow, the samples were broken much more often. This may indicate a greater rigidity and hardness of the material synthesized under such conditions.

**Figure 9:**
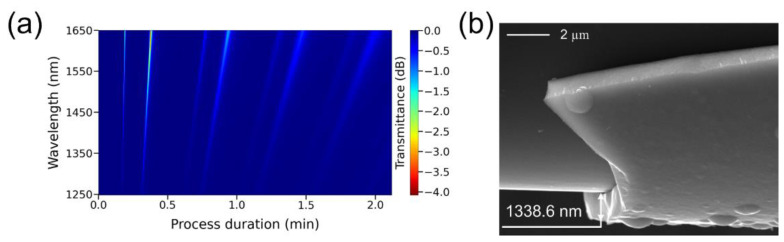
(**a**) Spectral response as a function of process duration for a thinned optical fiber with a ZnTe coating in the wavelength range of 1250–1650 nm. (**b**) SEM image of the destroyed layer.

To verify the accuracy of our method for determining the refractive index and thickness of films, we carried out an additional process of synthesis of the ZnTe coating. The thinned fiber region with a length of 2.6 mm and a diameter of 25.9 microns was located 11 mm from the furnace center in the direction of the gas flow, at a reactor temperature of 310 °C. Despite the change in the synthesis conditions, the film refractive index at a wavelength of 1450 nm, calculated by the method of neighboring resonances, like in the previous experiment, is 2.78. Using the FEM method and (1), we obtain that $\;d_{1\;TE}=43.9\;\mathrm{nm}$, $d_{1\;TM}=138.9\;\mathrm{nm}$, and ΔD=304.8 nm. Figure 7a shows that $d=d_{1\;TE}+4.26\cdot \Delta D$ or $d=d_{1\;TM}+3.94\cdot \Delta D$, the average value of which is 1340.7 nm. To determine the thickness of this layer on a scanning electron microscope, it was attached to an adhesive tape and pulled along the axis to break the film. The d value obtained from the SEM image was 1338.6 nm (Figure 9b), which confirms the high accuracy of the NLM for calculating the film thickness.

### 3.3. Dependence of the Growth Rate on the Outer Diameter of the Fiber

**Figure 10:**
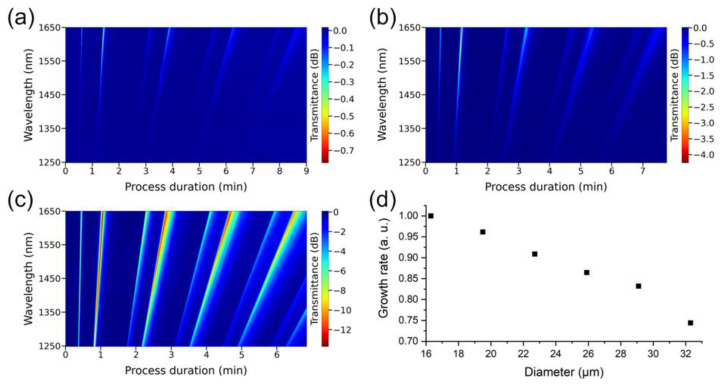
Spectral response in the wavelength range of 1250–1650 nm depending on the duration of the synthesis process of ZnTe film on the surface of a thinned optical fiber with an outer diameter of (**a**) 32.3 µm (**b**) 25.9 µm (**c**) 16.3 µm. (**d**) Dependence of the normalized rate of coating deposition on the outer diameter of the etched fiber region.

In addition to the calculation by the method of neighboring resonances, the thickness of the coating synthesized on the optical fiber surface was also estimated by counting the number of orders of interference fringes over the entire deposition zone. It appeared that the results of such estimates were almost two times lower than the outcomes of our calculations. To understand whether this is due to the different curvature radii of the lateral surfaces of the thinned and untouched regions of the fiber, an additional experiment was performed. A zinc telluride film was deposited on the surface of an etched section of a fiber light guide 2.4 mm long. The diameter of the thinned region successively changed from 32.3 to 16.3 μm in increments of about 3 μm (Figure 10). Obviously, with decreasing fiber diameter, the resonances became significantly deeper (Figure 10a–c). However, along with this, the time passing between the observation of neighboring pairs of resonances reduces; that is, the growth rate of the films enhances (Figure 10d). This effect of the influence of the fiber diameter on the growth rate of the film on its surface, according to our knowledge, was observed for the first time.

### 3.4. Manufacturing of Sensing Elements for Fiber-Optic Refractometers

**Figure 11:**
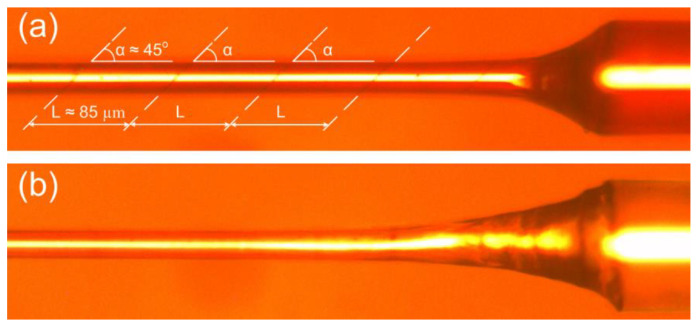
Photographs of sections of the thinned regions of optical fibers coated with ZnTe films of the thickness necessary to observe the first TM LMR in the near-infrared region. The layer is synthesized on the fiber (**a**) without and (**b**) with interruption of the process. The images are taken from different ends of the optical fiber etched section.

Due to the fact that the coefficient of thermal expansion (CTE) ZnTe [34] exceeds the CTE for SiO_2_ by more than an order of magnitude [35], periodic inclined cracks occur in the films at an angle of about 45° relative to the optical fiber axis. Such defects appear in films even at a thickness that allows observation in the near-infrared region (about 1000 nm) of the 1st TM LMR (Figure 11a). From a practical point of view, cracks in the sensor coating cause the test solution to seep under the film and eventually destroy it. As a result, the spectral position of the resonance varies from experience to experience, thus impairing the reproducibility of determining the environment refractive index. To eliminate this problem, we created an additional interface inside the zinc telluride layer by interrupting the film synthesis process. To accomplish this, approximately in the middle of the process, the supply of reagents to the reactor was interrupted for several minutes, after which the deposition of the film continued until a resonance appeared at the desired wavelength. This approach made it feasible to completely eliminate cracks in ZnTe coatings with a thickness sufficient to observe the first TM LMR in the near-infrared region spectral range. Both samples (Figure 11) were created on the same optical fiber with a thinned region 3.5 mm long and 23 µm in outer diameter.

**Figure 12:**
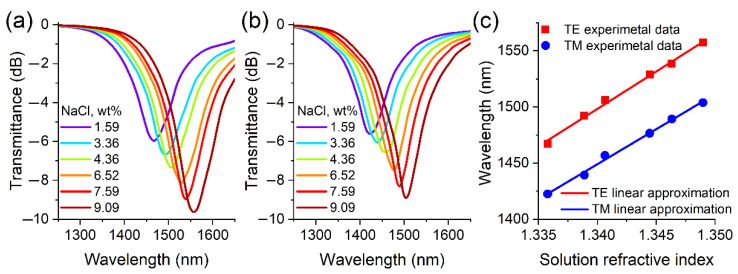
Transmission spectra of thinned optical fibers coated with zinc telluride when immersed in aqueous solutions of sodium chloride in the case of observation of the first (**a**) TE and (**b**) TM LMRs. (**c**) Dependences of the resonance maximum wavelength on the environment refractive index.

Before the first immersion of the optical fiber thinned part in water, the synthesized films were exposed to air at room temperature for at least 12 h. To measure the sensitivity of the sensors, aqueous solutions of table salt were used. The values of the refractive indices of the studied fluids (nD at a wavelength of 589 nm) were set on the basis of reference data [36]. Figure 12 shows that the sensitivity to changes in the refractive index in the investigated range is 6656 and 6240 nm/RIU for the first TE and TM LMR, respectively. According to our data, this is the highest value of sensitivity to changes in the NaCl solution refractive index among its counterparts (Table 1). Despite the fact that the sensitivity of the TE resonance is greater than the one of the TM LMR, the figure of merit (the ratio of sensitivity to FWHM) turned out to be larger in the second case (47 RIU^−1^) than in the first (42 RIU^−1^).

## 4. Conclusions

In this study, chemically thinned optical fibers coated with zinc telluride were manufactured. The influence of the temperature and the ratio of the initial reagents in the reaction zone on the form of LMR was investigated. Using only the transmission spectra obtained during the process, the refractive index of ZnTe films was calculated and the dependence of the growth rate on the distance to the center of the reactor was determined. It has been shown that with a decrease in the outer diameter of the thinned part of the fiber, the growth rate of the coating increases. The manufactured optical fibers were used as a sensitive part of fiber refractometers, the principle of operation of which is based on the dependence of the spectral position of the first TE and TM LMR on the refractive index of the environment. It was found that in the second case, due to the greater thickness of the film, they crack, which can be avoided by interrupting the coating process. The sensitivity of the sensors to changes in the refractive index of an aqueous sodium chloride solution in the range from 1.336 to 1.349 for the first TE and TM LMRS was 6656 nm/RIU and 6240 nm/RIU, respectively, which exceeds the previously presented LMR refractometers.

## Figures and Tables

**Table 1:** Comparison of sensitivity to NaCl concentration of fiber refractometers based on the LMR effect.

Coating Material	Refractive Index Range	Sensitivity (nm/RIU)	Ref.
TiO_2_	1.33–1.40	4122	[37]
SnO_2_	1.34–1.38	4704	[38]
Nb_2_O_5_	1.33–1.36	5072	[29]
ZnTe	1.33–1.35	6656	This work

## Data Availability

Not applicable.
